# Multiple-omics analysis of aggrephagy-related cellular patterns and development of an aggrephagy-related signature for hepatocellular carcinoma

**DOI:** 10.1186/s12957-025-03816-z

**Published:** 2025-04-30

**Authors:** Jiafen Xie, Xiaoming Wang

**Affiliations:** Department of Hepatobiliary Surgery, The First Affiliated Hospital of Guangzhou Medical University, 151 Yanjiang Xi Road, Yuexiu District, Guangzhou, 510120 China

**Keywords:** Aggrephagy, Hepatocellular carcinoma, Single-cell RNA-sequencing, Prognostic genes, Immunohistochemical analysis

## Abstract

**Background:**

Protein aggrephagy, a selected autophagy process response for degrading protein aggregates, plays a critical role in various cancers. However, its regulatory mechanisms and clinical implications in hepatocellular carcinoma (HCC) remain largely unexplored.

**Methods:**

We integrated bulk RNA-seq data from TCGA and single-cell RNA sequencing (scRNA-seq) data from GEO databases to systematically analyze aggrephagy-related genes (AGGRGs) in HCC. Prognostic aggrephagy-related genes (AGGRGs) were identified through univariate Cox and LASSO regression analyses, followed by the construction of a risk prediction model. Patients were stratified into high- and low-risk groups based on the median risk score. Comparative analyses were performed to assess clinical outcomes, pathway enrichment, and drug sensitivity. Independent risk factors were incorporated a nomogram using univariate and multivariate Cox regression. At the single-cell level, the AGG scores were calculated using AUCell algorithm, and cell interactions and pseudotime trajectory analyses were conducted. Finally, protein levels of key AGGRG was assessed via tissue microarray.

**Results:**

Eight AGGRGs (PFKP, TPX2, UBE2S, GOT2, ST6GALNAC4, ADAM15, G6PD, and KPNA2) were identified as prognostic markers for HCC. The high-risk group exhibited significantly worse survival outcomes, heightened drug resistance, and enrichment in cell cycle, mTORC1 signaling, and reactive oxygen species pathways. Single-cell transcriptomic analysis revealed 11 distinct cell types within the HCC tumor microenvironment (TME), including hepatocytes, T cells, NK cells, macrophages, monocytes, dendritic cells, plasma B cells, mature B cells, mast cells, endothelial cells, and fibroblasts. Hepatocytes exhibited the highest AGGRG scores and were associated with metabolic reprograming, proliferation, and immune evasion. Further subclustering of malignant hepatocytes using inferCNV revealed eight functionally heterogeneous subpopulations with extensive intercellular crosstalk. Trajectory analysis showed G6PD- and CCNB1-expressing subpopulations in early-to-intermediate differentiation states, whereas C3 and ARGs marked terminal differentiation. Notably, G6PD was predominantly expressed in early and mid-stages, while KPNA2, PFKP, and TPX2 were upregulated in advanced tumor states. Immunohistochemical (IHC) validation confirmed significant overexpression of G6PD in HCC tissues compared to adjacent normal tissues.

**Conclusion:**

These findings provide a molecular framework for targeting aggrephagy pathways in HCC treatment strategies.

**Supplementary Information:**

The online version contains supplementary material available at 10.1186/s12957-025-03816-z.

## Introduction

Liver cancer is the third leading cause of cancer-related deaths, with approximately 865,000 new cases and 757,948 deaths reported in 2022. Hepatocellular carcinoma (HCC) accounts for over 80% of primary liver cancers [1]. The primary etiological factors driving HCC development include chronic hepatitis B virus (HBV) and hepatitis C virus (HCV) infections, which contribute to hepatocarcinogenesis [2]. Additional risk factors encompass aflatoxin exposure, heavy alcohol consumption, smoking, excess body weight, and metabolism disorders such as non-alcoholic steatohepatitis (NASH) and type 2 diabetes mellitus [2, 3]. Although the widespread use of the HBV vaccine has reduced HBV infections [4], and improvements in early detection and therapeutic strategies have enhanced the prevention efforts [5], high recurrence rates and poor five-year survival outcomes remain as major clinical challenges [6]. Therefore, elucidating the molecular mechanisms underlying HCC pathogenesis and identifying novel prognostic biomarkers are imperative for developing more effective therapeutic interventions and improving patient survival.

The ubiquitin–proteasome system (UPS) and autophagy are the two primary cellular pathways responsible for maintaining cellular homeostasis, or proteostasis, by mitigating the harmful effects of unfolded, misfolded, or damaged proteins [7]. When the proteasome function is impaired, aggrephagy (AGG), a selective autophagic process that clears potentially toxic protein aggregates [8]. Growing evidence implicates that AGG plays a critical role in various pathological conditions, including cancer [9]. In tumorigenesis, AGG plays a particularly intriguing role.

Notably, p53 mutant aggregation drives oncogenesis through multiple mechanisms, including loss of function, dominant-negative effects, and gain-of-function effects [10]. Targeting p53 protein aggregation has been proposed as a potential anticancer therapeutic strategy [11]. Recent work by Sun et al. further highlighted the clinical relevance of AGG by characterizing its role in the tumor microenvironment (TME) of lung adenocarcinoma (LUAD) and identifying AGG-related prognostic markers [12]. Despite these advances, the functional significance and regulatory mechanisms of AGG in HCC remain poorly understood.

This study employed a multi-omics approach, integrating bulk RNA sequencing (RNA-seq) and single-cell RNA sequencing (scRNA-seq) analysis to systematically in HCC pathogenesis and their potential as prognostic biomarkers. Finally, we employed immunohistochemical (IHC) analysis to confirm the protein levels of key AGGRGs in HCC tumors, thereby bridging the gap between bioinformatic predictions and clinical relevance.

## Methods and statistical analysis

### Data collection

The RNA-seq data, clinical information, and single nucleotide mutation data for TCGA-LIHC cohort (comprising 371 HCC tumor samples and 50 normal samples) were collected from the UCSC Xena website (https://xenabrowser.net/) and used as training dataset. mRNA expression profiles of 81 HCC tumor samples and corresponding clinical data were obtained from Gene Expression Omnibus (GEO) under accession GSE54236 and used as a validation dataset to assess the risk model. Additionally, mRNA expression profiles of 247 HCC tumor samples from GEO database under accession GSE14520 and used as a validation dataset to evaluate the expression of the key genes. Single-cell RNA-seq (scRNA-seq) data for primary and/or metastatic tumor tissues and adjacent non-tumor livers were obtained from 10 patients with HCC by accessing GSE149614 in GEO database [13]. Additionally, a total of 1,368 Aggrephagy-related genes (AGGRGs) were downloaded from the GeneCards Human Gene Database (GeneCards, https://www.genecards.org/).

### Identification of the differentially expressed genes (DEGs) and selection of aggrephagy-related genes (AGGRGs) based on bulk RNA-seq data

The differentially expressed genes (DEGs) between HCC tumor samples and normal samples from TCGA-LIHC cohort were identified using DESeq2 package (version 1.38.2) with the thresholds of log2 |fold change (FC)| > 1 and *p*-value < 0.05. The differentially expressed AGGRGs (DE-AGGRGs) were obtained by intersecting DEGs and 1,368 AGGRGs from GeneCards database (Table S1).

### Construction and evaluation of a AGG-related prognostic model

Univariate Cox regression analysis was performed using “survival” package to obtain the survival-related AGGRGs with the criteria of *p*-value < 0.05. These AGGRGs were shrunk using least absolute shrinkage and selection operator (LASSO) regression using “glmnet” package. The selected prognostic AGGRGs were visualized using a forest plot generated by the “forestplot” package. Hazard ratio (HR) > 1 represents risk factor, and HR < 1 represents protective factor. Subsequently, risk score was calculated according to the formula, risk score =$\:\sum\:_{i=1}^{n}(expi\times\:\beta\:i)$, where expi represents expression level of each gene, and βi represents coefficient of corresponding gene. Patients with HCC in both the training set (TCGA-LIHC cohort) and validation set (GSE54236 cohort) were stratified into high-risk and low-risk groups based on the median of risk score. Kaplan-Meier (KM) survival curves were drawn using the “survminer” package, and the survival differences between high-risk and low-risk groups were determined using the log-rank test. Finally, the receiver operating characteristic (ROC) curves for 1- and 3-year were generated using “survivalROC” package, and the area under the curve (AUC) value was calculated to assess the predictive performance of the prognostic model.

### Analysis of clinical characteristics, functional enrichment, drug sensitivity between high- and low-AGG risk score groups

Differences in clinical characteristics (race, age, gender, and stage) between high-risk and low-risk groups were evaluated using the chi-squared test. K-M curves stratified by clinical characteristics (age, gender, and stage) were generated to assess survival differences between the high-risk and low-risk groups. Functional enrichment analysis between high-risk and low-risk groups was performed using the “GSVA” package, with differential pathway enrichment assessed between the two groups using the “Limma” package. Furthermore, the drug sensitivity between high-risk and low-risk groups was predicted using “oncoPredict” package, based on Genomics of Drug Sensitivity in Cancer 2 (GDSC2, https://www.cancerrxgene.org/compounds) database [14].

### Construction and evaluation of a predictive nomogram

The risk score and clinical characteristics (race, age, gender, and stage) were integrated into the univariate and multivariate Cox regression analyses to select the independent risk factors using “survival” package. A predictive nomogram was constructed and visualized using “rms” and “regplot” packages base on the identified independent risk factors. A calibration curve was generated using “rms” package to evaluate the accuracy of the nomogram. The ROC curves were used to evaluate the sensitivity of the nomogram. And the decision curve analysis (DCA) curve was constructed to evaluate the clinical significance of the nomogram.

### Validation of the expression of prognostic genes in external dataset

The mRNA expression of prognostic genes in HCC tumor samples and normal samples was evaluated in GSE14520 dataset. K-M curves were generated to assess survival differences between the high-expression and low-expression group for each prognostic gene. Additionally, ROC curves were constructed to assess the sensitivity of each prognostic gene.

### Quality control and cell annotation in scRNA-se Q data

In the present study, Seurat package (version 4.3.0) was used to scRNA-seq data analysis. After excluding three samples (HCC07P, HCC08P, and HCC10L), 10 HCC tumor samples and 8 adjacent non-tumor samples were used for subsequent analyses. First, Low-quality cells were filtered based on the following criteria: fewer than 3 transcripts per cell, fewer than 200 or more than 7,000 expressed genes, or more than 20% mitochondrial genes. Finally, 71,050 cells and 25,479 genes were retained for further analysis. The scRNA-seq data were then normalized using “NormalizeData” function. The normalized data were transformed into Seurat objects, and the top 2,000 highly variable genes (HVGs) were selected using “FindVariableFeatures” function. Principle component analysis (PCA) was performed for linear dimension reduction using “RunPCA” function based on 2,000 HVGs. Batch correction and integration of the 18 samples were carried out using the “harmony” package. Dimensionality reduction and visualization was performed using uniform manifold approximation and projection (UMAP) with the “RunUMAP” and “DimPlot” functions. Finally, cell clustering was performed using the “FindNeighbors” and “FindClusters” functions, followed by manual annotation to identify different cell clusters.

### Aggrephagy-related signature score

The AGG-score for each cell was calculated based on the expression profiles of AGGRGs using “AUCell” package. Cells were then classified into high-AGG and low-AGG score groups based on the median AUC value for further analysis.

### Functional enrichment analysis

Pathway enrichment between the high-AGG and low-AGG score groups was assessed using Gene set variation analysis (GSVA) with the “GSVA” package. Differential pathway enrichment between the two groups was determined using “limma” package. Additionally, the expression patterns of prognostic AGGRGs across different cell types were analyzed.

### Copy number alteration (CNV) inference

The CNV score for hepatocytes was assessed using “inferCNV” R package. K-means clustering was applied to differentiate malignant from non-malignant hepatocytes.

### Nonnegative matrix factorization (NMF) clustering in hepatocytes

The “NMF” package was used to extract cluster top loading features and perform dimensionality reduction analysis on hepatocytes based on the scRNA-seq expression matrix [15]. The optimal number of clusters was determined using the cophenetic coefficient.

### Cell-to-cell communication

The “CellChat” package was used to analyze intercellular interactions in scRNA-seq data [16]. In the present study, we focused on the communication dynamics between high AGG-score group and low AGG-score group, as well as among the different hepatocyte subpopulations. The “netAnalysis_contribution” function was utilized to assess the contribution of the ligand-receptor interactions within the intercellular communication networks. Furthermore, key cellular pathways influenced by these interactions were identified to provide insights into the underlying molecular mechanisms.

### Pseudotime trajectory analysis

Pseudotime trajectory analysis was performed using the “Monocle 2” package to elucidate the cellular evolution patterns of hepatocytes in relation to AGG-score. The top 2,000 HVGs were selected as the features genes. Dimensionality reduction was conducted using the “reduceDimension” function, and the trajectory was constructed and visualized using “plot_cell_trajectory” function. The “DDRtree” algorithm was applied to infer pseudotime progression and cellular differentiation states. Additionally, the expression dynamics of prognostic AGGRGs in hepatocytes were analyzed to reveal their trends across pseudotime.

### Validation of the protein expression of G6PD in HCC

To investigate the expression of G6PD protein in HCC, we analyzed data from the Human Protein Atlas (https://www.proteinatlas.org/) to compare its expression in HCC tumor tissues and normal liver tissues. Subsequently, tissue microarray (TMA) and immunohistochemistry (IHC) were performed to validate the expression of G6PD protein in HCC tumor tissues and adjacent non-tumor tissues. The TMA of HCC samples was purchased from Shanghai Xinchao Biotechnology Co. and included 166 HCC samples (83 tumor tissues and their adjacent non-tumor counterparts). The microarray sections were incubated overnight at 4 °C with a primary antibody anti-G6PD (1:600, ab210702, Abcam, MA, USA), followed by incubation with a secondary antibody for 1 h at room temperature. Finally, the samples were stained with DAB and hematoxylin for visualization. To quantitatively assess G6PD expression, the histological score (H-score) was calculated based on staining intensity and coverage. The H-score ranged from 0 to 300, determined using the formula, H-score =$\:\sum\:$ (I × Pi), where Pi represents the percentage of positively stained tumor cells, and I denotes the staining intensity [17].

## Results

### Construction and evaluation of an AGG-related prognostic model

**Fig. 1:**
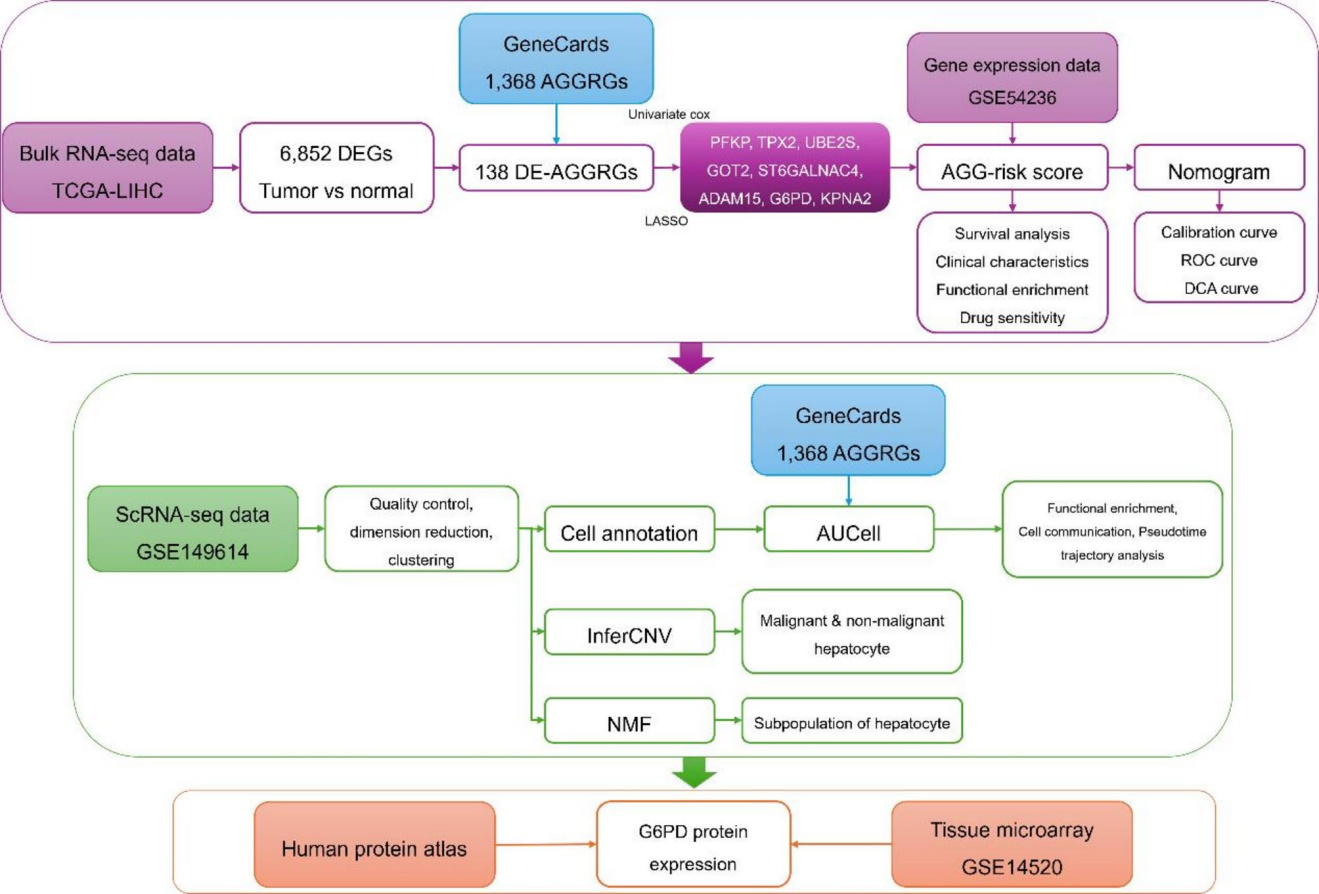
Workflow chart of the this study

In the represent study, we systematically integrated the gene expression data, scRNA-seq data, and protein expression data to investigate the prognostic significance and molecular characteristics in HCC. The overall study design is illustrated in the workflow chart (Fig. 1).

**Fig. 2:**
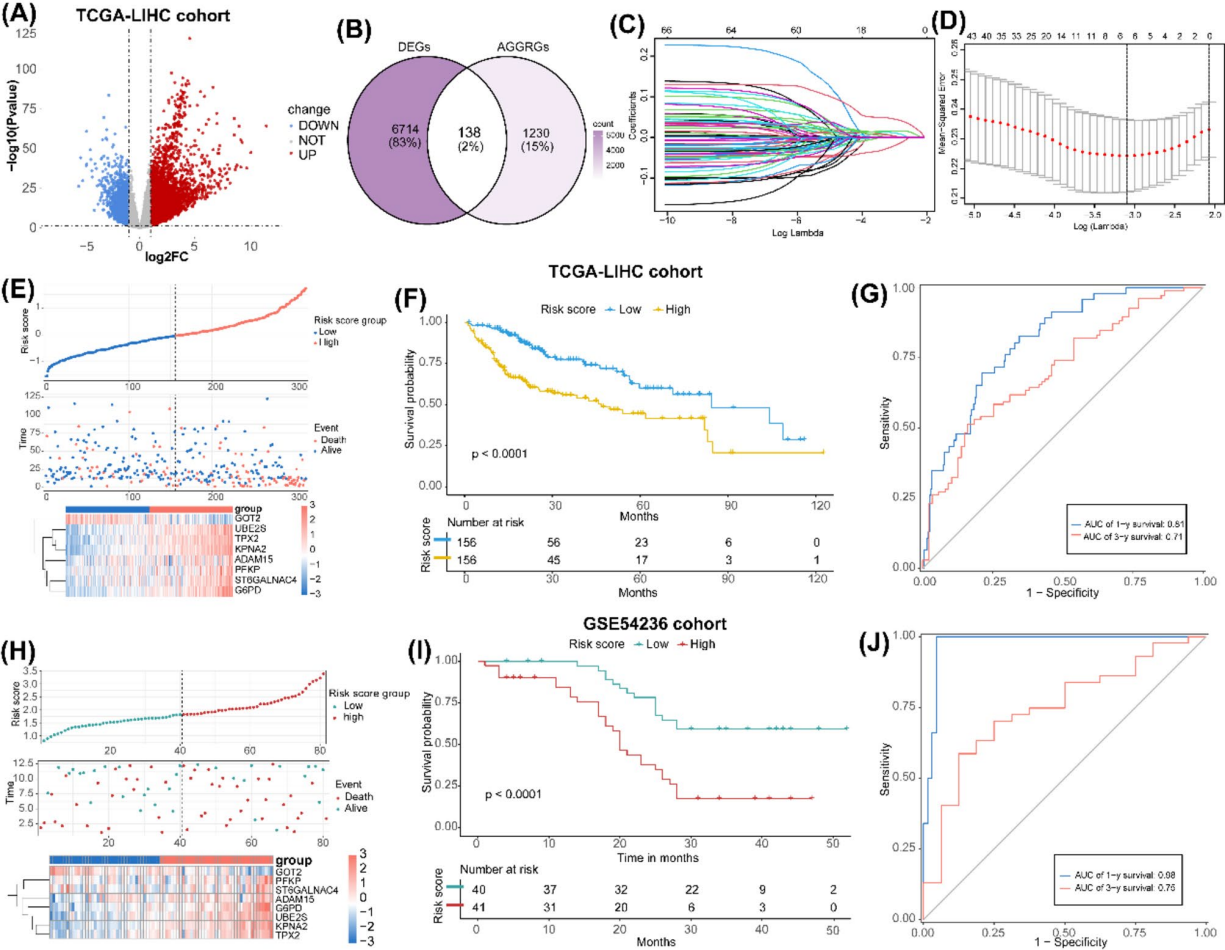
Construction and Evaluation of an AGG-Related Prognostic Model. (**A**) The volcano plot of DEGs between tumor samples and normal samples in the TCGA-LIHC dataset with the thresholds of log2 |FC| > 1 and *p*-value < 0.05. (**B**) The Venn diagram of differentially expressed AGGRGs by interesting DEGs of TCGA-LIHC dataset and 1,368 AGGRGs from GeneCards database. (**C**) The plots of coefficient distribution of log (lambda) of LASSO regression model. (**D**) Selection of the optimal parameters (lambda) of LASSO regression model. (**E**) and (**H**) Upper: Patients in (**E**) TCGA-LIHC cohort and (**H**) GSE54236 cohorts were divided into high-risk and low-risk groups according to median value of risk score. Medium: Survival states of patients in (**E**) TCGA-LIHC cohort and (**H**) GSE54236 cohorts. Bottom: Heatmap of the expression of prognostic genes in (**E**) TCGA-LIHC cohort and (**H**) GSE54236 cohorts. (**F**) and (**I**) Kaplan − Meier survival curves of the survival states between high-risk and low-risk groups in (**F**) TCGA-LIHC cohort and (**I**) GSE54236 cohorts. (**G**) and (**J**) Time-dependent ROC curves for 1- and 3-year survival of patients in (**G**) TCGA-LIHC cohort and (**J**) GSE54236 cohorts

First, a total of 6,852 DEGs (5,275 upregulated and 1,577 downregulated) were identified between tumor and normal samples in TCGA-LIHC cohort (Fig. 2A, Table S2). Subsequently, 138 differentially expressed AGGRGs (DE-AGGRGs) were yielded by intersecting 6,852 DEGs and 1,368 AGGRGs obtained from GeneCards (Fig. 2B, Table S3).

Next, 138 AGGRGs were incorporated into univariate Cox (Table S4) and LASSO regression analyses to select the prognostic genes (Fig. 2C-D). Eight AGGRGs (PFKP, TPX2, UBE2S, GOT2, ST6GALNAC4, ADAM15, G6PD, and KPNA2) were identified as the prognostic genes. Then, the AGG-related risk score was calculated based on eight prognostic genes to construct a risk model. Patients in both TCGA-LIHC and GSE54236 cohorts were stratified into high-risk and low-risk groups according to the median of risk score (Fig. 2E and H). K-M survival analysis indicated that significantly worse clinical outcomes in high-risk patients compared to their low-risk counterparts in both cohorts (Fig. 2F and I).

To further evaluate the predictive performance of our model, we generated time-dependent ROC curves. The model demonstrated excellent prognostic capability, with area under the curve (AUC) values of 0.81 and 0.71 for 1- and 3-year survival prediction in the TCGA-LIHC cohort, respectively (Fig. 2G). Similarly, in the GSE54236 cohort, the model achieved outstanding AUC values of 0.98 and 0.75 for 1- and 3-year survival prediction (Fig. 2J). These findings suggest that our AGG-related risk model exhibits robust performance in survival prediction, particularly for short- to medium-term (1–3 years) prognosis in HCC patients.

### Analysis of clinicopathological characteristics, functional enrichment, and drug sensitivity between high- and low-AGG risk score groups

**Fig. 3:**
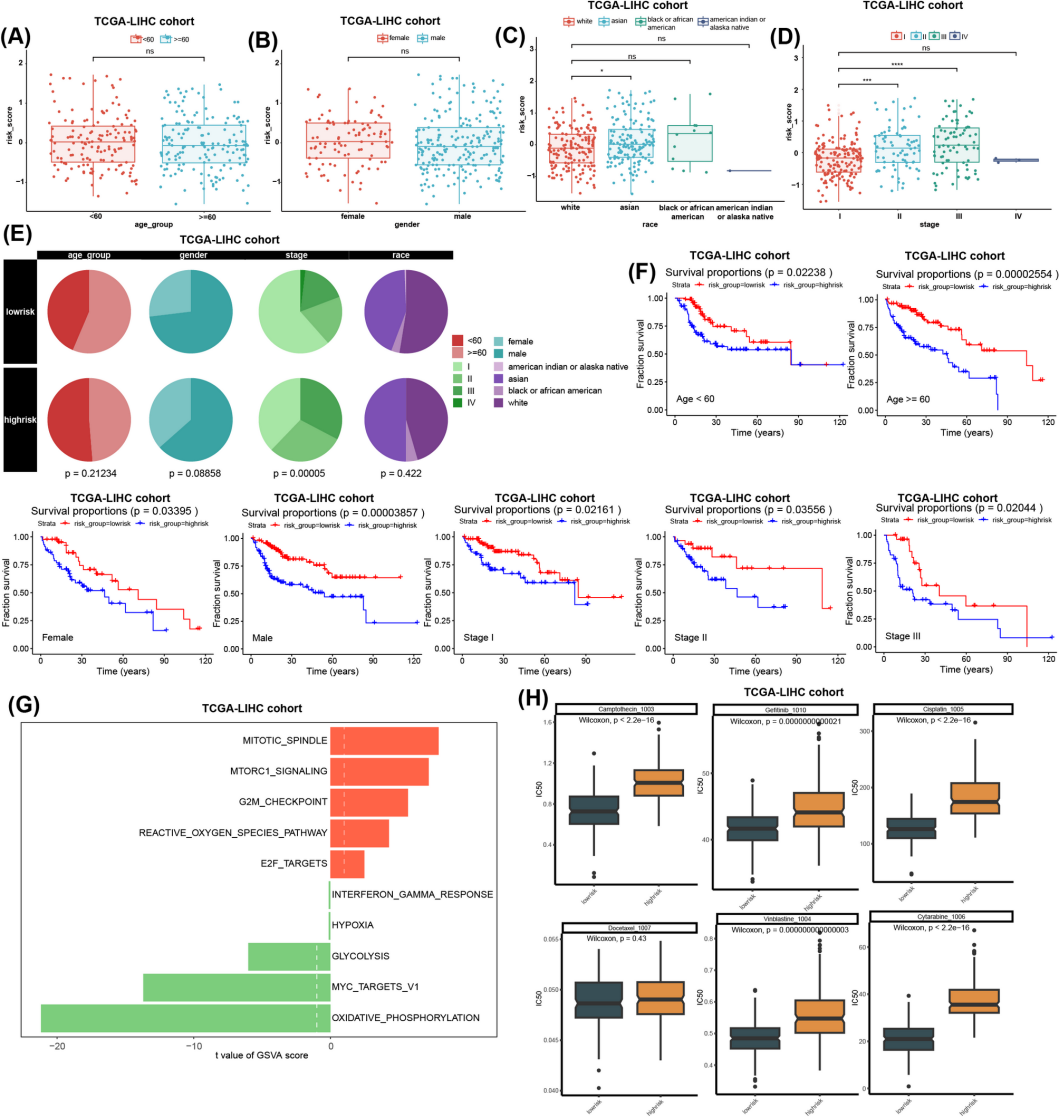
Analysis of clinicopathological characteristics, functional enrichment, and drug sensitivity between high- and low-agg risk score groups. (**A**)-(**D**) Boxplots of differences in clinicopathological characteristics (age, gender, race, and clinical stages) between high-risk and low-risk groups in TCGA-LIHC cohort. (**E**) Pie chart of differences in clinicopathological characteristics (age, gender, race, and clinical stages) between high-risk and low-risk groups in TCGA-LIHC cohort. (**F**) Kaplan − Meier survival curves of the survival states between high-risk and low-risk groups in TCGA-LIHC cohort, patients stratified with clinicopathological characteristics (age, gender, race, and clinical stages). (**G**) Bar plots of the differentially enriched pathways between high-risk and low-risk groups in TCGA-LIHC cohort. (**H**) Boxplots of differences in half maximal inhibitory concentration (IC50) value between high-risk and low-risk groups in TCGA-LIHC cohort

To further characterize the clinical relevance of our prognostic model, we explored the association between AGG-related risk scores and the clinicopathological characteristics in the TCGA-LIHC cohort. While no significant differences in age, gender, and race between high-risk and low-risk groups, we observed the notable differences in clinical stages, with higher-risk patients tending to present with more advanced stages (Fig. 3A-E). Stratified survival analysis demonstrated that elevated risk scores were consistently associated with poorer clinical outcomes across all subgroups, indicating the robustness of our model independent of other clinicopathological factors (Fig. 3F). Moreover, functional enrichment analysis revealed distinct biological pathways associated with each risk groups (Fig. 3G). High risk score group exhibited significant enrichment in cell cycle-related pathways (mitotic spindle, G2M checkpoint, E2F targets) and mTORc1 signaling pathway, and reactive oxygen species (ROS) pathway. In contrast, the low-risk group showed predominant activation of interferon gamma (IFN-γ) response, hypoxia, glycolysis, MYC target v1, and oxidative phosphorylation. Furthermore, drug sensitivity demonstrated that high-risk patients displayed significant resistance to multiple chemotherapeutic agents, including Camptothecin, Gefitib, Cisplatin, Docetaxel, Vinblastine, and Cytarabine (Fig. 3H). These findings suggest that the AGG-related risk score not only serves as a robust prognostic indicator but also reflects distinct molecular characteristics and treatment responses in HCC patients.

### Development of a predictive nomogram

**Fig. 4:**
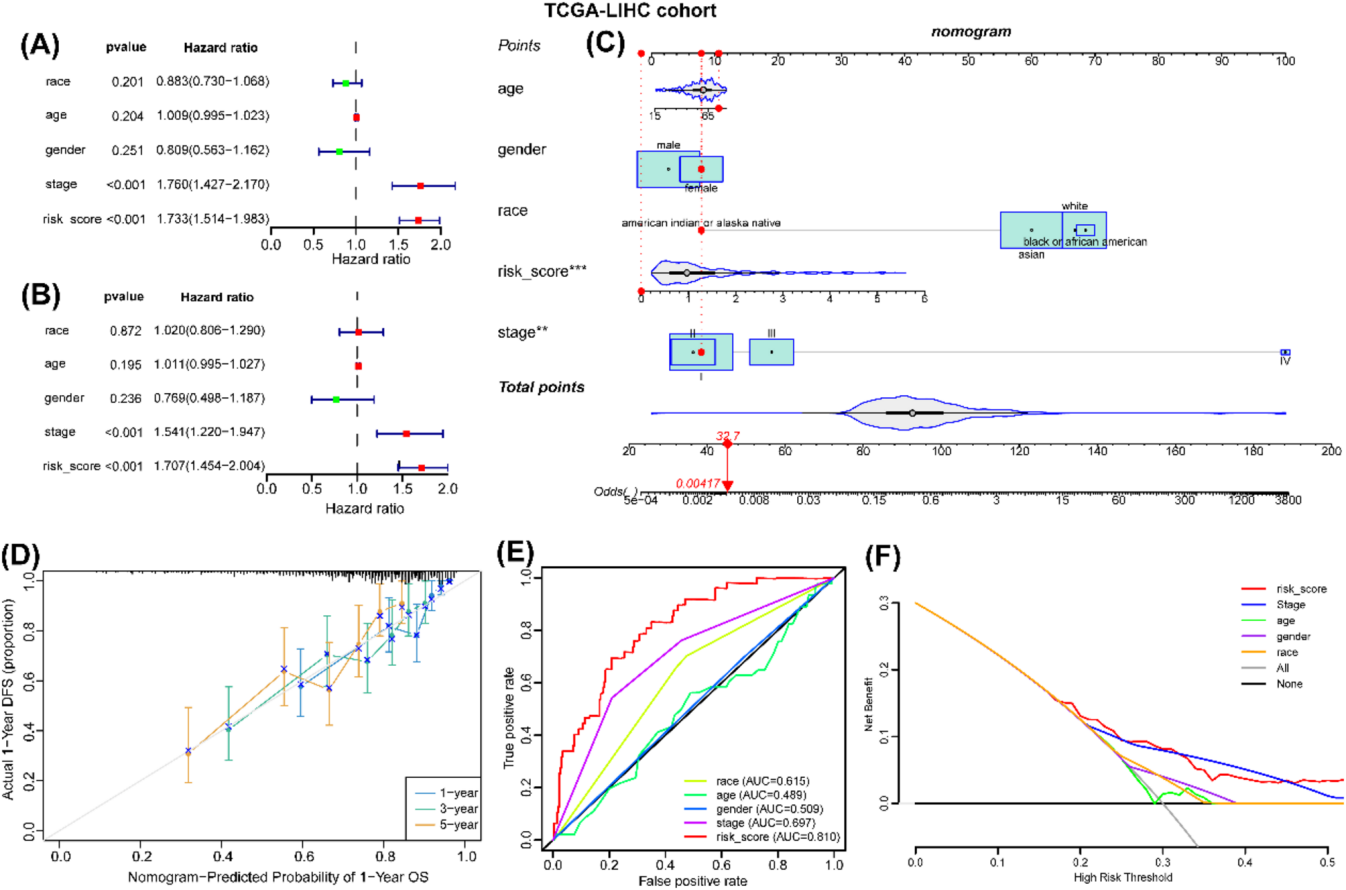
Development of a Predictive Nomogram. (**A**)-(**B**) Forest plots of univariate and multivariate Cox regression analyses in TCGA-LIHC cohort by incorporating risk score and clinicopathological characteristics (age, gender, race, and clinical stages). (**C**) Nomogram of predicting of overall survival for HCC patients in TCGA-LIHC cohort. (**D**) Calibration curve, (**E**) decision curve, and (**F**) ROC curve for nomogram

To further assess the clinical utility of our prognostic model, the univariate and multivariate cox regression analyses were performed to identify the independent factors (Fig. 4A-B). The AGG-related risk score and clinical stages were identified as independent factors (Fig. 4A-B). Then, we construct a predictive nomogram based o the independent factors (Fig. 4C). The calibration curve analysis indicated the concordance between observation and prediction, suggesting that the excellent predictive accuracy of the nomogram (Fig. 4D). The predictive performance was further validated through ROC analysis, the AUC value of 0.697 for clinical stage, and 0.81 for risk score, which indicated the superior sensitivity of the nomogram (Fig. 4E). The DCA curve revealed that the nomogram and risk score were more stable and accurate in accurate prognostic predictions compared to using clinical stage alone (Fig. 4F). These findings suggest that our nomogram serves as a robust tool for individualized prognosis prediction in HCC patients.

### Single-cell characterization of the tumor microenvironment (TME) in HCC

**Fig. 5:**
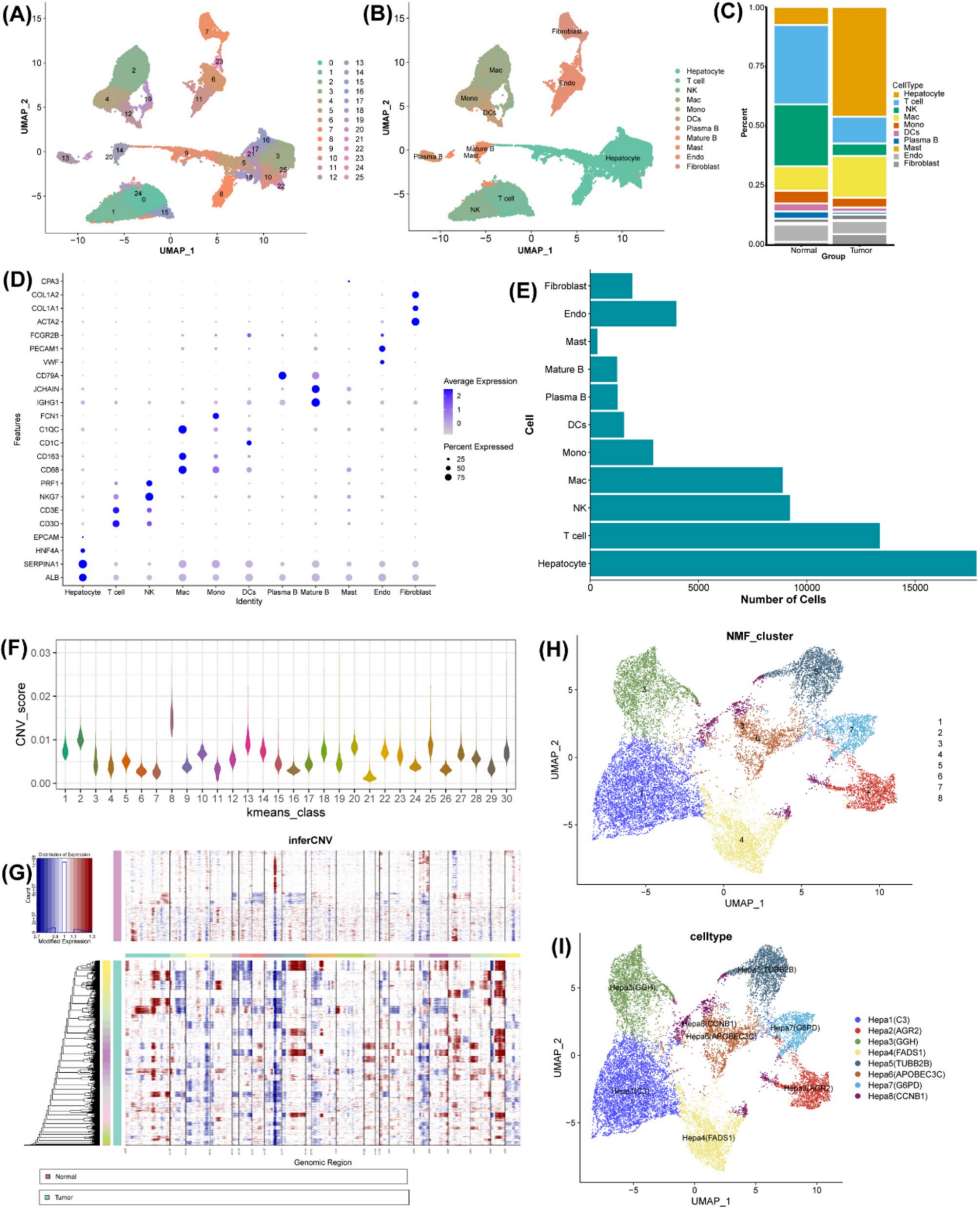
Single-Cell Characterization of the Tumor Microenvironment (TME) in HCC. (**A**) UMAP plots of 26 cell clusters. (**B**) UMAP plots of distribution of 11 annotated cell populations. (**C**) Histogram of the percentage of cell types between tumor samples and normal samples. (**D**) Bubble plots of the top 3 gene markers for each cell type. (**E**) Histogram of the numbers of cell types across different samples. (**F**) Violin plots of the CNV score for hepatocytes across different samples. (**G**) Heatmap of the CNV patterns in hepatocytes across different samples. (**H**) UMAP plots of 8 cell clusters of hepatocytes. (**I**) UMAP plots of distribution of 8 re-clustered sub-groups of hepatocytes

We further characterized the cell landscape of HCC by performing high-resolution single-cell RNA-sequencing analysis of the TME. Following rigorous quality control, a total of 71,050 cells and 25,479 genes were retained for further analysis (Figure S1A-D). After sample integration and batch effects correction (Figure S1E-F), unsupervised clustering revealed that cells were clustered into 26 distinct cellular subgroups (Figure S1G, and Fig. 5A). Subsequently, eleven major cell clusters were manually annotated according to the known markers for each cell populations (Fig. 5B and D), including hepatocyte, T cell, natural Killer (NK) Cell, macrophage (Mac), monocyte (Mono), dendritic cells (DCs), plasma B, mature B, mast cell, endothelial cell (Endo), and fibroblast. As shown in Fig. 5C, we observed significant compositional differences between tumor and normal samples. Generally, we found the increased proportions of hepatocyte, mast cell, and fibroblast, while the decreased infiltration of other cell types in tumor samples compared to normal samples. Notably, hepatocytes contributed to the predominant cellular component in HCC TME (Fig. 5C and E). The CNV analysis demonstrated that significantly elevated CNV scores in tumor-derived hepatocytes compared to their normal counterparts, conforming their malignant transformation (Fig. 5F and G). Then, eight distinct hepatocyte subpopulations were identified using NMF clustering methods (Fig. 5H-I). This refined classification revealed previously unappreciated diversity within the malignant hepatocyte compartment, suggesting potential functional specialization among tumor cells.

### Identification of the AGG-related cell populations

**Fig. 6:**
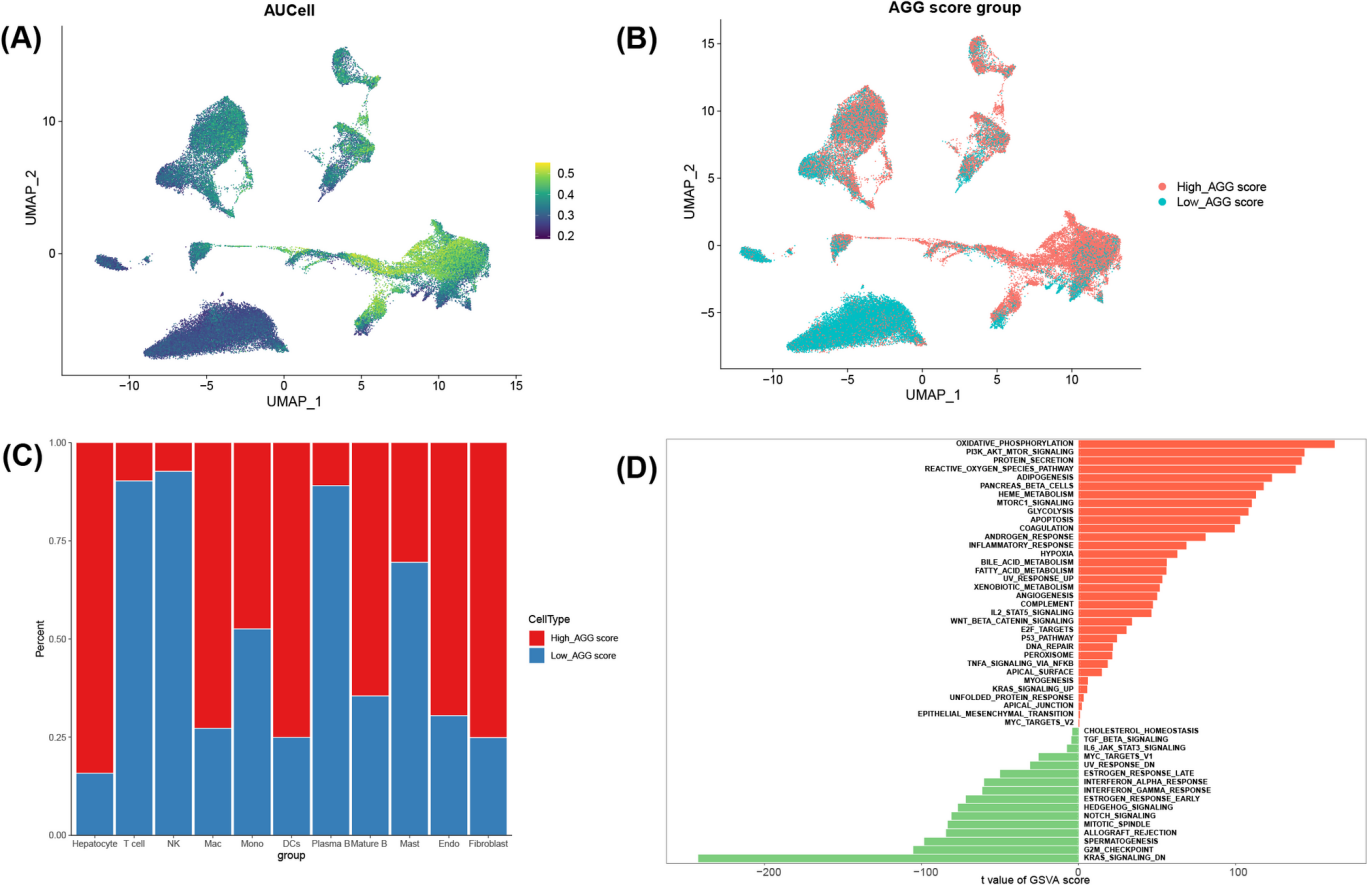
Identification of the AGG-related cell populations. (**A**) UMAP plots of distribution of cell populations with AUCell score based on the expression profiles of AGGRGs. (**B**) UMAP plots of distribution of cell population between high- and low-AUCell score based on the median value of the AUC. (**C**) Histogram of the percentage of cell types between high- and low-AUCell score groups. (**D**) Bar plots of the differentially enriched pathways between high- and low-AUCell score groups

To investigate the AGG activity at single-cell solution, AUCell algorithm was used to calculate the AGG-score for each cell (Fig. 6A), all cells were distributed into high- and low-AGG score groups (Fig. 6B), revealing that shrinking cellular heterogeneity within the TME. Hepatocytes were identified as the predominant AGG-active population, comprising more than 75% of high-AGG score cells (Fig. 6C).

In addition, functional enrichment analysis revealed that high-AGG score cells were involved in cell cycle (E2F targets and MYC targets V2), metabolism reprograming (bile acid and fatty acid metabolism), and immune evasion mechanisms (wnt-β/catenin, and TNFs/NF-кB signaling pathways) (Fig. 6D). While, the low-AGG score cells were involved in metabolic regulation (cholesterol homeostasis) and inflammatory response (TGF-β, and IL6/JAK/STAT3 signaling pathways) (Fig. 6D). These results demonstrate that AGG activity defines functionally distinct cellular states within the HCC ecosystem. The predominance of hepatocytes in the high-AGG compartment particularly implicates malignant hepatocytes as key drivers of AGG-related pathophysiology in HCC.

### Analysis of cell-to-cell communication and cell-state transition trajectories with AGG score

**Fig. 7:**
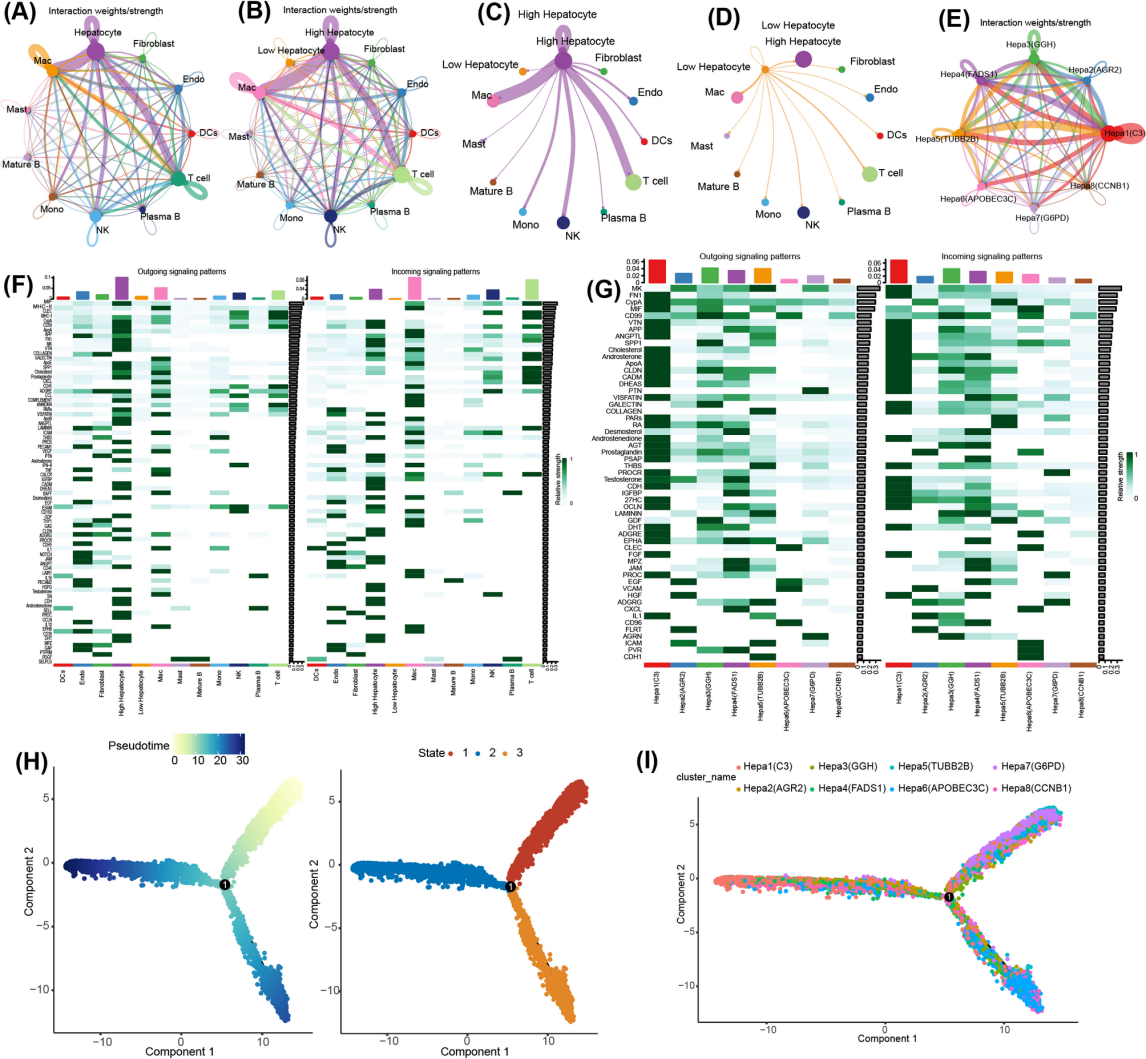
Analysis of Cell-to-Cell Communication and Cell-State Transition Trajectories with AGG Score. (**A**) The network of the number and weight of communications across different cell types. (**B**) The network of the number and weight of communications between high- and low-AUCell score groups. (**C**) The network of the number and weight of communications between high-AUCell score hepatocytes and other cell types. (**D**) The network of the number and weight of communications between low-AUCell score hepatocytes and other cell types. (**E**) The network of the number and weight of communications across different subtypes of hepatocytes. (**F**) Heatmap of signaling pathways communications across different cell types. (**G**) Heatmap of signaling pathways communications between high- and low-AUCell score groups. (**H**) Pseudo-time and cell trajectory analysis for hepatocytes. (**I**) Cell trajectory analysis for subpopulation of hepatocytes

Through comprehensive evaluation of ligand-receptor pair expression patterns, we systematically characterized the spectrum of interactions among cellular clusters (Fig. 7A). Additionally, we investigated the communication patterns between high-AGG score hepatocytes and their low-AGG score counterparts in terms of interaction intensity and partner diversity (Fig. 7B). Our findings revealed that high-AGG score exhibited significantly more extensive and stronger interactions with various cell types compared to low-AGG score hepatocytes (Fig. 7C-D). The heatmap visualization of signaling directionality identified 77 distinct signaling pathways, which contributed to the enhanced communication capacity of high-AGG score hepatocytes (Fig. 7E). We further observed a broadcast-type communication pattern among hepatocyte subpopulations (Fig. 7F). The detailed analysis revealed that particularly robust interaction networks centered around hepa1 (C3) and hepa3 (GGH) subtypes (Fig. 7G). These findings suggest that AGG was involved in mediating both hepatocyte-extrinsic communications and intrinsic inter-subpopulation interactions.

We further performed the pseudotime trajectory analysis to investigate the differentiation dynamics of high-AGG score hepatocyte (Fig. 7H), the results revealed that hepatocytes were categorized into three states (state1/2/3). The trajectory of subpopulations of hepatocytes indicated that hepa7 (G6PD) and hepa8 (CCNB1) corresponded to early and intermediate cellular phases, while hepa1 (C3) and hepa2 (AGR2) associated with advanced and terminal cellular phases (Fig. 7I). These results reflect a gradual transition from an early state of metabolic activity and rapid proliferation towards functional maturation, accompanied by substantial transcriptional reprogramming along the pseudotime axis.

### Single-cell validation of the prognostic AGGRGs expression

**Fig. 8:**
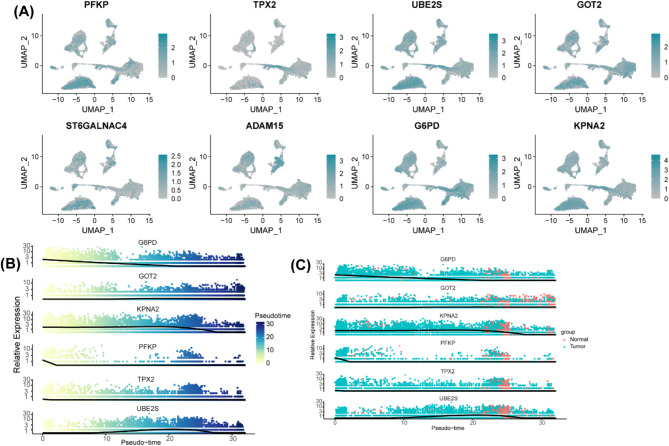
Single-Cell Validation of the Prognostic AGGRGs Expression. (**A**) UMAP plots of each prognostic gene expression in different cell populations. (**B**) The expression dynamics of prognostic genes are differentially expressed across pseudotime. (**C**) The expression dynamics of prognostic genes are differentially expressed across pseudotime between tumor samples and normal samples

We have established the prognostic significance of AGGRGs at transcriptome level, we next explored their expression patterns at single-cell level. We observed that widespread expression of these genes across hepatocyte populations (Fig. 8A), with particularly prominent expression of GOT2 and G6PD in hepatocytes. Pseudotime projections analysis of transcriptional dynamics demonstrated distinct temporal expression patterns among prognostic genes. Upregulation of G6PD was observed in early cellular phases, while upregulation of KPNA2, PFKP, and TPX2 were observed in advanced and terminal cellular phases (Fig. 8B). Comparative analysis between tumor and normal samples revealed fundamental differences in developmental expression patterns (Fig. 8C). We observed more heterogeneous gene expression distributions in normal samples, whereas pronounced phase-specific expression peaks in tumor samples, which were G6PD and KPNA2 exhibited significant expression peaks in early cellular phases, but KPNA2, PFKP, UBE2S, and TPX2 showed higher expression levels in the advanced and terminal cellular phases in the tumor samples. These results suggested that these genes exhibit tumor-specific expression dynamics during cellular differentiation, potentially contributing to tumor progression and influencing patient outcomes.

### Validation of G6PD expression in HCC

**Fig. 9:**
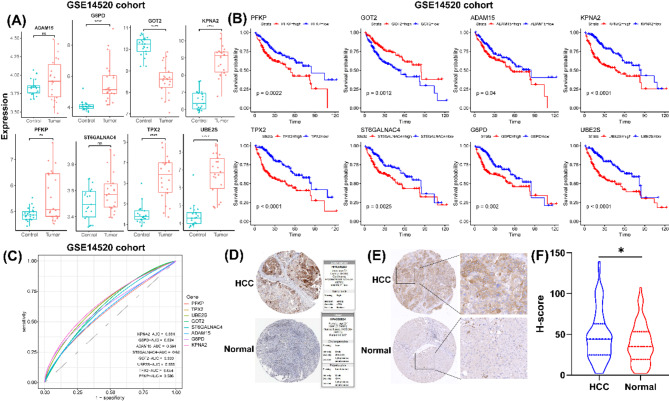
Validation of G6PD Expression in HCC. (**A**) Box plots of the expression of prognostic genes between tumor samples and normal samples in GSE14520 dataset. (**B**) Kaplan − Meier survival curves of the survival states between high- and low- expression of each prognostic gene. (**C**) ROC curves for each gene expression. (**D**) IHC staining images of G6PD expression between tumor sample and normal sample, source from Human protein atlas (HPA). (**E**) IHC staining images of G6PD expression between tumor samples and adjacent normal samples from tissue microarray (Scar bar = 100 μm & 200 μm)

To clinically validate our findings, we first explored the expression patterns of prognostic genes in an external dataset (GSE14520). As shown in Fig. 9A, we found the increased expression of PFKP, TPX2, UBE2S, ST6GALNAC4, ADAM15, G6PD, and KPNA2, but decreased expression of GOT2 in tumor samples compared to normal samples. High expression of PFKP, TPX2, UBE2S, ST6GALNAC4, ADAM15, G6PD, and KPNA2 associated with poor survival states, but high expression of GOT2 associated with favorable survival states (Fig. 9B). To assess the predictive sensitivity of these genes, the AUC value for each gene was evaluated. The AUC 0.586 for PFKP, 0.654 for TPX2, 0.655 for UBE2S, 0.62 for ST6GALNAC4, 0.594 for ADAM15, 0.624 for G6PD, 0.664 for KPNA2, and 0.633 for GOT (Fig. 9C). These results underscore the potential of these genes as prognostic biomarkers for HCC. Given the crucial role of G6PD in HCC progression. As identified through transcriptomic and single-cell analyses, we further examined its protein expression. In IHC staining, we observed representative images of G6PD expression in HCC tumor samples and normal samples (Fig. 9D-E). Notably, G6PD expression was significantly elevated in tumor samples compared to normal tissues (Fig. 9F). These findings supported the potential role of G6PD in HCC pathogenesis.

## Discussion

The accumulation of protein aggregates is a hallmark of various pathologies and plays a central role in the development of several human diseases [18], including neurodegeneration [19], muscle disorders [20], Preeclampsia [21], diabetes [22], and cancer [23]. The selective degradation of protein aggregates through AGG is essential for maintaining cellular proteostasis [24]. For example, preventing the aggregation of mutant p53 proteins has emerged as a promising cancer treatment strategy, aiming to inhibit their misfolding and accumulation [11]. Increasing evidence highlights the critical role of AGG in cancer, with several emerging therapeutic strategies targeting this process for anti-tumor interventions. However, the precise role and underlying mechanisms of aggregate autophagy in HCC remain largely unexplored. Therefore, understanding the role of AGGRGs and their regulatory mechanisms may provide novel insights and promising therapeutic targets for HCC.

In the current study, we investigated the prognostic value of AGGRGs and the impact of AGG on TME in HCC by integrating bulk RNA-seq and scRNA-seq data. First, we identified eight prognostic AGGRGs (PFKP, TPX2, UBE2S, GOT2, ST6GALNAC4, ADAM15, G6PD, and KPNA2) as prognostic signatures and calculated the AGG-related risk score using univariate Cox and LASSO regression analyses. Patients with high-risk scores exhibited significantly poorer survival outcomes in both the training and validation cohorts. Additionally, high-risk patients showed increased drug resistance, further suggesting an unfavorable prognosis. These findings underscore the critical prognostic significance of AGGRGs. Subsequently, we conducted an in-depth investigation into the mechanisms by which AGG influences the TME in HCC, providing further insights into its potential role in disease progression and therapeutic resistance.

Based on scRNA-seq data, we identified eleven major cell types in TME of HCC, including hepatocyte, T cell, NK Cell, Mac, Mono, DCs, plasma B, mature B, mast cell, Endo, and fibroblast. Among these, hepatocytes were the most abundant cell type in the TME. As primary parenchymal cells of the liver, hepatocytes play critical roles in innate immunity, metabolism, detoxification, and protein synthesis [25]. In HCC tumor samples, malignant hepatocytes with high CNV scores have been identified. Further analysis revealed eight distinct hepatocyte subpopulations were identified, including hepa1 (C3), hepa2 (AGR2), hepa3 (GGH), hepa4 (FADS1), hepa5 (TUBB2B), hepa6 (APOBEC3C), hepa7 (G6PD), and hepa8 (CCNB1).

Hepa1 (C3) is a subpopulation expresses complement C3. C3 is a central effector molecule of the complement system [26]. C3 has been identified as a novel biomarker in non-alcoholic fatty liver disease (NAFLD) [27], and is involved in cell migration, invasion, and epithelial-mesenchymal transition (EMT) in HCC [28]. hepa2 is characterized by AGR2 (anterior gradient-2) expression, this subgroup demonstrates strong association with metastatic potential. High expression of AGR2 correlates with unfavorable patient outcomes [29, 30]. hepa3 has been defined as GGH (γ-glutamyl-hydrolase) expression. GGH is a ubiquitously expressed enzyme and participates in cell proliferation, DNA synthesis and repair [31]. Its expression is linked to poor clinical outcome of tumors [31, 32]. hepa4 is a subpopulation, expresses FADS1 (fatty acid desaturase-1). FADS1 is a rate-limiting enzyme and relates to biosynthesis of long-chain polyunsaturated fatty acids [33]. FADS1 acts as an oncogene to promote tumor growth [34]. hepa5 is marked by TUBB2B (tubulin beta class I genes) expression and links to tumor growth, immune infiltration, drug resistance, and lipid metabolism dysregulation [35, 36]. hepa6 is a subpopulation that expresses APOBEC3C (Apolipoprotein B mRNA-editing enzyme catalytic polypeptide-like 3 C). APOBEC3C mediates tumor immunomodulation and stemness maintenance across various malignancies [37]. hepa7 has been defined by G6PD (Glucose-6-phosphate dehydrogenase), which is an enzyme crucial for energy metabolism. G6PD plays an essential role in cell proliferation, survival, and stress responses, particularly in cancer contexts [38, 39]. Hepa8 has been characterized by CCNB1 (cyclin B1). CCNB1 is a key regulator of the cell cycle and serves as a master regulator of mitotic progression [40, 41]. This comprehensive characterization of hepatocyte subpopulations provides critical insights into the cellular heterogeneity and functional diversity within the HCC tumor ecosystem, highlighting potential therapeutic targets and prognostic markers for clinical application.

We further found that hepatocytes exhibited the highest AGG score among all cell types in the HCC microenvironment. Subsequent cell-to-cell communication analysis revealed that high-AGG score hepatocytes established significantly more extensive and stronger interactions with other cell populations compared to low-AGG counterparts. Notably, among hepatocyte subpopulations, hepa1 (C3) and hepa3 (GGH) emerged as major communication hubs, forming dense interaction networks with other cell types. Pseudotime trajectory analysis of high-AGG hepatocytes revealed a clear differentiation pattern. hepa7 (G6PD) and hepa8 (CCNB1) represented early and intermediate cellular phases, while hepa1 (C3) and hepa2 (AGR2) corresponded to advanced and terminal phases. Expression profiling demonstrated that all eight prognostic AGGRGs were abundantly expressed in hepatocytes, with GOT2 and G6PD showing particularly prominent expression patterns.

Additionally, dynamic analysis along the differentiation trajectory revealed that G6PD expression was elevated in the early cellular phases, while PNA2, PFKP, and TPX2 were elevated in the advanced and terminal phases. Compared to normal samples, G6PD and KPNA2 exhibited significant expression peaks in the early cellular phases in tumor samples, and more homogeneous expression distribution was displayed in normal samples. These findings suggest that G6PD and KPNA2 may server as potential biomarkers for early diagnosis, prognosis prediction, and patients stratification in HCC. Therefore, we further investigated the expression of AGGRGs and their clinical relevance in a independent cohort. External validation using the GSE14520 cohort confirmed the prognostic significance of AGGRGs, with G6PD showing consistent clinical relevance. Immunohistochemical validation at the protein level demonstrated significant G6PD overexpression in HCC tissues compared to normal controls, corroborating previous study linking elevated G6PD contributes to cell migration and invasion, as well as poor prognosis of HCC [42].

In our study, we comprehensively integrate the single-cell transcriptomic data, bulk RNA-seq data, and protein expression data to explore the prognostic value of AGG in HCC. We identified cell subpopulations associated with AGG and elucidated the role of prognostic genes in cell differentiation and tumor progression. Although our study provides important insights into AGG in HCC, our study has certain limitations. Future research will focus on further validating the mechanisms of AGGRGs in HCC using various approaches, including in vitro cell experiments and in vivo animal models.

## Conclusion

In conclusion, our study is the first to discover the role and underlying mechanisms of AGG in HCC, and to identify the potential promising diagnostic and treatment targets for HCC.

## Electronic supplementary material

Below is the link to the electronic supplementary material.

Supplementary Material 1: Figure S1. Single-cell RNA-sequencing data processing

Supplementary Material 2: Table S1 Aggrephagy-related gene from GeneCards.

Supplementary Material 3: Table S2 Differentially expressed genes between tumor samples and normal samples in RCGA-LIHC cohort.

Supplementary Material 4: Table S3 The differentially expressed AGGRGs by interesting DEGs of TCGA-LIHC dataset and 1,368 AGGRGs from GeneCards database.

Supplementary Material 5: Table S4 Univariate Cox regression analysis of the survival-related AGGRGs with the criteria of p-value < 0.05.

## Data Availability

The datasets used and analyzed in this study are publicly available from the TCGA and GEO databases. Additional details are provided in the Methods section.
