# Supplementary material for: Multiple-omics analysis of aggrephagy-related cellular patterns and development of an aggrephagy-related signature for hepatocellular carcinoma
Source: World J Surg Oncol. 2025 Apr 30;23:175. doi: 10.1186/s12957-025-03816-z (PMC12044776; doi:10.1186/s12957-025-03816-z)
Supplement: Supplementary file 1 — Supplementary Material 1: Figure S1. Single-cell RNA-sequencing data processing [file 12957_2025_3816_MOESM1_ESM.docx]

**
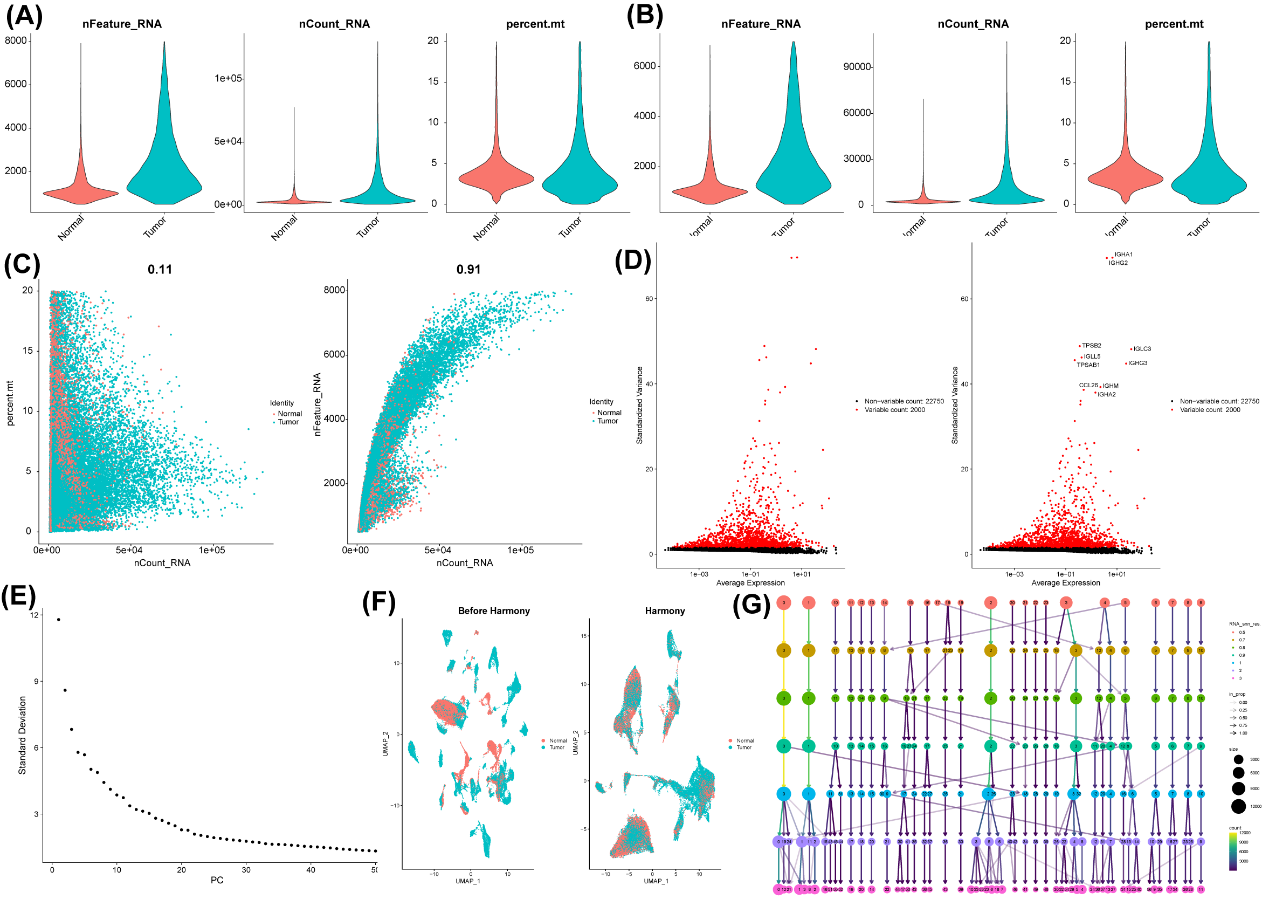
**

**Figure S1. Single-cell RNA-sequencing data processing.**

(A)-(C) Violin plots of before and after of Single-cell quality control. Single cell gene count is between 200-7000, nCount_RNA is 200 or more, and mitochondrial percentage is below 20%.

(D)-(E) The volcano plot of top 2,000 highly variable genes (HVGs).

(E) The distribution of the top 50 ranked principal components (PCs).

(F) UMAP plots of the all clusters across different samples before and after harmony.

(G) Clustree of clustering at optimal resolutions (0.8).
